# Use of Erector Spinae Plane Block for Perioperative Pain Control in a Patient Undergoing Spinal Surgery

**DOI:** 10.7759/cureus.9646

**Published:** 2020-08-10

**Authors:** Ruben Schwartz, Ivan Urits, Omar Viswanath, Alan D Kaye, Jonathan Eskander

**Affiliations:** 1 Anesthesiology, Mount Sinai Medical Center of Florida, Miami, USA; 2 Anesthesia, Critical Care and Pain Medicine, Beth Israel Deaconess Medical Center, Harvard Medical School, Boston, USA; 3 Pain Management, Valley Pain Consultants - Envision Physician Services, Phoenix, USA; 4 Anesthesiology, Louisiana State University Health Sciences Center, Shreveport, USA; 5 Anesthesiology, Portsmouth Anesthesia Associates, Portsmouth, USA

**Keywords:** dexmedetomidine, dexamethasone, erector spinae plane block, anterior cervical discectomy fusion

## Abstract

Regional anesthetic techniques have become a vital part of the perioperative pain control process. The opioid crisis remains a major obstacle in the medical field today and many practitioners have looked upon regional nerve blocks to decrease opioid usage. The erector spinae plane block (ESPB) has gained prominence as a viable option for perioperative pain control for numerous procedures. Spinal surgery, although mostly utilized to relieve back pain, can be extremely painful for the patient perioperatively. To mitigate pain, many practitioners have turned to oral analgesics as regional techniques have not been typically employed. Anterior cervical discectomy and fusion (ACDF) surgeries in particular have been implicated as exquisitely painful and may predispose patients to sustained opioid use postoperatively. Many of these patients are on chronic opioid therapy and they have developed the syndrome of opioid abuse hyperalgesia; therefore, decreasing the need for opioids postoperatively is of utmost importance. We present the case of a successful ESPB performed prior to emergence for a patient undergoing ACDF to limit opioid consumption. Informed consent was provided by the patient for this case report.

## Introduction

One of the greatest fear patients have when they come for surgery is pain. Postoperative pain control is of utmost importance to improve a patient's chance of a successful recovery and potential discharge from the hospital. Anesthesiologists play a vital role in the perioperative pain control process. Regional anesthetic techniques have been demonstrated as crucial to control pain after surgery and limit opioid consumption. One technique in particular, the erector spinae plane block (ESPB) has been shown to help with pain after breast, thoracic, and abdominal surgeries [1]. It has been utilized in coronary artery bypass surgery as well [2]. Spinal surgery remains as one of the more painful operations with minimal regional techniques typically employed [3]. Anterior cervical discectomy and fusion (ACDF) surgeries in particular have been implicated as painful and may predispose patients to long-term opioid use postoperatively [4]. The goal for patients after surgery is to control pain in a safe way by limiting opioid consumption as much as possible. Systemic opioids have been shown to be extremely detrimental causing respiratory depression, reintubation, atelectasis, constipation, among others. In this case report we describe the use of an ESPB for a patient undergoing ACDF to successfully limit opioid requirements postoperatively. 

## Case presentation

A 67-year-old African American male with past medical history of benign prostatic hypertrophy and erectile dysfunction presented for C6-T1 ACDF due to cervical radiculopathy. An ESPB block was performed intraoperatively. Prior to emergence from anesthesia, the patient was placed in the lateral decubitus position. Using sterile technique and ultrasound guidance, a single shot ESPB was performed bilaterally at the T1 level. A sterile ultrasound probe was utilized to visualize the fascial planes. Using an in-plane approach the probe was placed vertically at the T1 level and the transverse process was initially identified. The transverse process can be seen as a "squared off" structure deep to the fascial planes. Next, a 4 cm 21-gauge stimuplex needle was introduced and the transverse process was initially contacted and the needle withdrawn superficially. The fascial plane was hydrodissected in a caudal direction and the injectate was administered. Initially, 40 mL of 0.2% ropivicaine was mixed with 10 mg of preservative-free dexamethasone and 50 mcg of dexmedetomidine (Dex-Dex). Some 20 mL of the Dex-Dex solution was deposited in the erector spinae plane on each side for a total of 40 mL administered.

 After the block, the patient was successfully extubated and transferred to the postanesthesia care unit (PACU) in stable condition. In the PACU he reported 2/10 pain and required no opioids. He had neither motor deficits nor hemodynamic instability. He had no difficulty swallowing solids and acknowledged numbness over the C3-T3 region. Over the course of the next three days he reported his pain as 0/10. He required no pain medications until postoperative day 4, on which he required oxycodone 5 mg - acetaminophen 325 mg once in the morning and once at night. At this time, sensory blockade began to diminish, though he reported no further opioid use in the subsequent days. As reported by the patient, his sensory blockade lasted about four days.

## Discussion

The ACDF surgery, although routinely performed by many spinal surgeons, is a technically demanding procedure with rare but serious complications including respiratory insufficiency, dysphagia, and recurrent laryngeal nerve injury [5-6]. These problems are most often caused by retraction of structures in the neck for visualization by the surgeon. These potential catastrophic events would be further exacerbated by opioid use and further respiratory depression. It is of utmost importance to decrease narcotic requirements for all surgeries but especially those within the neck.

## Conclusions

Enhanced recovery after surgery (ERAS) protocols have been instrumental in curbing perioperative opioid use throughout the nation. Regional anesthetic techniques are one of the mainstays of these regimens providing multi-modal analgesia for surgical patients. The ESPB is a viable and valuable addition to the anesthetic plan for ACDF surgeries that could be a part of ERAS protocols for these operations in the future. Further large studies are needed to assess the safety and efficacy of the ESPB for ACDF.
